# Study on the prediction of short-term clinical changes after cerebral infarction using carotid plaque ultrasound strain elastography

**DOI:** 10.3389/fneur.2025.1652157

**Published:** 2025-09-04

**Authors:** Mingqiu Li, Shiwen Weng, Qian Song, Huiyu Ge

**Affiliations:** ^1^Department of Ultrasound, Beijing Chaoyang Hospital, Capital Medical University, Beijing, China; ^2^Department of Neurology, Beijing Tiantan Hospital, Capital Medical University, Beijing, China

**Keywords:** carotid plaque, ultrasound strain elastography, cerebral infarction, clinicaldeterioration, short-term prognosis

## Abstract

**Objective:**

To evaluate the value of ultrasound strain elastography in assessing carotid plaque stiffness for predicting short-term clinical changes after cerebral infarction.

**Methods:**

Patients with cerebral infarction and carotid atherosclerotic plaque identified through routine ultrasound examination at the Ultrasound Department of Beijing Chaoyang Hospital, Capital Medical University, were selected for this study. All patients underwent strain elastography. Based on changes in their clinical conditions within 30 days following cerebral infarction, they were divided into a deterioration group and a non-deterioration group. The differences between the two groups in terms of strain elastography results were compared for statistical significance. Logistic regression analysis was conducted to analyze factors affecting short-term clinical changes in patients with cerebral infarction. The receiver operating characteristic (ROC) curve was drawn.

**Results:**

A total of 110 patients were included in this study (83 males and 27 females, average age: 60.02 ± 10.67 years). The carotid plaque strain elastography value, arterial wall strain elastography value and plaque stiffness were1.17 ± 0.40, 0.53 ± 0.16 and 2.33 ± 0.97 in the deterioration group, 1.73 ± 0.58, 0.59 ± 0.18 and 3.04 ± 1.00 in the non-deterioration group. The differences between the two groups were statistically significant about carotid plaque strain elastography value and plaque stiffness. The AUC values for predicting non-deterioration after cerebral infarction were 0.790 for carotid plaque strain elastography value, 0.608 for arterial wall strain elastography value, and 0.740 for plaque stiffness.

**Conclusion:**

Carotid plaque ultrasound strain elastography can be utilized to assess short-term clinical changes after cerebral infarction. Patients who experience clinical deterioration have lower carotid plaque strain elastography values, indicating that this parameter is more effective in predicting the absence of deterioration after cerebral infarction.

## 1 Introduction

Cerebral infarction is a major cause of permanent disability, while vulnerable carotid plaques are a key factor in the occurrence and progression of acute cerebral infarction (1, 2). Studies have shown that vulnerable carotid plaques are an independent risk factor for the onset and recurrence of cardiovascular and cerebrovascular diseases. The thickness, size, surface morphology and internal neovascularization, and so on of carotid plaques are typically associated with the occurrence and recurrence of ischemic cardiovascular and cerebrovascular events (3, 4). While strain elastography is of certain value in assessing the occurrence of cerebral infarction, few studies have explored its role in predicting short-term clinical changes following cerebral infarction 5–9). In this study, by comparing carotid plaque strain elastography findings of patients with different short-term clinical changes after cerebral infarction, we aim to determine its effectiveness in evaluating short-term clinical changes in patients with cerebral infarction.

## 2 Materials and methods

### 2.1 Research objects

Patients diagnosed with non-cardiogenic cerebral infarction who underwent carotid ultrasound examination at Beijing Chaoyang Hospital, Capital Medical University between February 2022 and December 2023 were selected for this study. All patients underwent both routine ultrasound and ultrasound strain elastography examinations within 3 days. All patients received standardized neurology treatment after cerebral infarction. Based on changes in their clinical conditions within 30 days after cerebral infarction, they were divided into a deterioration group and a non-deterioration group. The study had been approved by the Ethics Committee of Beijing Chaoyang Hospital, Capital Medical University and all participants had provided written informed consent prior to ultrasound examination.

Inclusion criteria: (1) age ≥ 18 years; (2) first-ever ischemic cerebral infarction affecting the anterior circulation within the past 30 days; (3) at least one non-hyperechoic carotid plaque on the side ipsilateral to the cerebral infarction; (4) no ≥50% stenosis in intracranial arteries, extracranial carotid arteries, or vertebral arteries on imaging.

Exclusion criteria: (1) prior history of neck radiotherapy; (2) diagnosed with cardiogenic cerebral infarction or cerebral infarction of undetermined etiology based on the TOAST classification; (3) unable to cooperate with the ultrasound examination due to consciousness disorders, soft tissue infections in the neck; (4) poor quality of ultrasound images caused by obesity and other reasons; (5) previous carotid endarterectomy or stent placement; (6) refusal to participate in this study; (7) loss to follow-up.

### 2.2 Instruments and technique

In this study, a LogiqE9 color Doppler ultrasound system manufactured by GE, equipped with strain elastography software and analysis software was employed. Probe selection: a 9 L linear array probe with a frequency of 5–10 MHz was selected. The gain was adjusted to ensure clear signals without noise. The dynamic range (DR) was set between 55 and 65% to clearly display the plaque and surrounding tissues. The focus point was placed in the region of interest (ROI) or slightly distally to it.

#### 2.2.1 Routine ultrasound examination

The patients took a supine position with a thin pillow behind the neck, and the head was tilted backward and turned away from the examination side by about 45° to fully expose the neck. A routine grayscale ultrasound examination was performed to record the number of plaques, as well as the location, length and thickness of the largest non-hyperechoic plaque.

#### 2.2.2 Real-time ultrasound strain elastography examination

Based on the routine ultrasound results, real-time ultrasound strain elastography was conducted. The probe was gently moved to center the target plaque on the screen, and the distance between the plaque and the surface of the probe ranges from 1.0 cm to 3.0 cm. After that, the elastography software was activated. Real-time grayscale ultrasound image and strain elastography image were displayed side by side. The sampling frame was adjusted, so that the entire plaque, as well as the anterior and posterior walls of the vessel, can be included within the sampling frame and remained stationary. Strain elastography was applied based on the pressure generated by vascular pulsation. The strain elastographic characteristics of plaques were observed, and both dynamic and static images were recorded for analysis. The examination was repeated three times in each patient. During the examination, pressure and compression rate were strictly maintained within the green range, with each recording lasting 3 s.

#### 2.2.3 Strain elastography analysis

The carotid plaque region was defined as Region A and the mean strain value across the plaque was recorded as plaque elasticity (A) of this patient. The carotid arterial wall region was defined as Region B and its strain value was recorded as carotid arterial wall elasticity (B). The ratio of A/B was calculated as the plaque stiffness. Each subject’s target plaque was measured three times and the average value was used for analysis.

After undergoing ultrasound examination, the patients were followed up for 30 days and their clinical changes after cerebral infarction were assessed. They were then divided into a deterioration group and a non-deterioration group based on the presence or absence of clinical deterioration. Deterioration refers to the progression of disease within 30 days following the initial episode of cerebral infarction. This may manifest as a newly detected infarct or an increase in infarct size on CT or MRI, and an elevated NIHSS score compared to baseline.

### 2.3 Statistical analysis

Data analysis was performed using SPSS 22.0. Measurement data that followed a normal distribution, including age, plaque size, and strain value, were expressed as x ± s. Comparisons between two independent samples were conducted using the independent samples t-test. Enumeration data were compared using the x^2^ test. Logistic regression was applied to analyze the influence of carotid plaque elastography results on the disease changes after cerebral infarction. The ROC curve and the area under the curve (AUC) were applied to determine the predictive power of carotid plaque elasticity for clinical changes after cerebral infarction, with *p* < 0.05 indicating statistically significant.

## 3 Results

### 3.1 Baseline demographics

A total of 116 patients diagnosed with cerebral infarction between February 2022 and December 2023 were selected for this study, 3 of whom were screen failures, 3 of whom were lost to follow-up. Ultimately, 110 patients were included in the final analysis, consisting of 83 (75.5%) males and 27 (24.5%) females with an average age of 60.02 ± 10.67 years. Among them, 87 (79.1%) had hypertension, 54 (49.1%) had diabetes, 97 (88.2%) had dyslipidemia, and 64 (58.2%) had a history of smoking. Based on clinical changes after cerebral infarction, 48 patients were categorized into the deterioration group and 62 were categorized in the non-deterioration group. No significant differences were observed between the two groups in terms of baseline demographics (Table 1).

**Table 1:** Comparison of baseline demographics between the deterioration group and the non-deterioration group.

Baseline characteristics	Deterioration group	Non-deterioration group	Test value	*p*-value
Number of Cases	48	62		
Gender (Male/Female)	39 (81.3%)/9 (18.7%)	44 (71.0%)/18 (29.0%)	1.54	0.21
Age (Years)	58.60 ± 10.91	61.11 ± 10.43	1.23	0.22
Smoking (Yes/No)	29 (60.4%)/19 (39.6%)	35 (56.5%)/27 (43.5%)	0.18	0.68
Diabetes (Yes/No)	30 (62.5%)/18 (37.5%)	33 (53.2%)/29 (46.8%)	0.95	0.33
Hypertension (Yes/No)	38 (79.2%)/10 (20.8%)	49 (79.0%)/13 (21.0%)	0.00	0.98
Dyslipidemia (Yes/No)	42 (87.5%)/6 (12.5%)	55 (88.7%)/7 (11.3%)	0.04	0.85

### 3.2 Basic characteristics of plaques

There were no significant differences between the deterioration group and the non-deterioration group in plaque length, thickness, echogenicity or surface morphology (*p* > 0.05; Table 2).

**Table 2:** Basic characteristics of plaques in the deterioration group and the non-deterioration group after cerebral infarction.

Characteristics	Deterioration group	Non-deterioration group	Test value	*p*-value
Number of cases	48	62		
Plaque length (mm)	12.22 ± 5.32	14.64 ± 7.48	1.89	0.06
Plaque thickness (mm)	2.46 ± 0.82	2.64 ± 0.87	0.34	0.74
Echogenicity (hypoechoic/isoechoic/mixed echoic)	1 (2.1%)/19 (39.6%)/28 (58.3%)	2 (3.2%)/20 (32.3%)/40 (64.5%)	0.71	0.70
Surface morphology (smooth/irregular)	11 (22.9%)/37 (77.1%)	13 (21.0%)/49 (79.0%)	0.06	0.81

### 3.3 Strain elastographic characteristics of plaques

**Figure 1:**
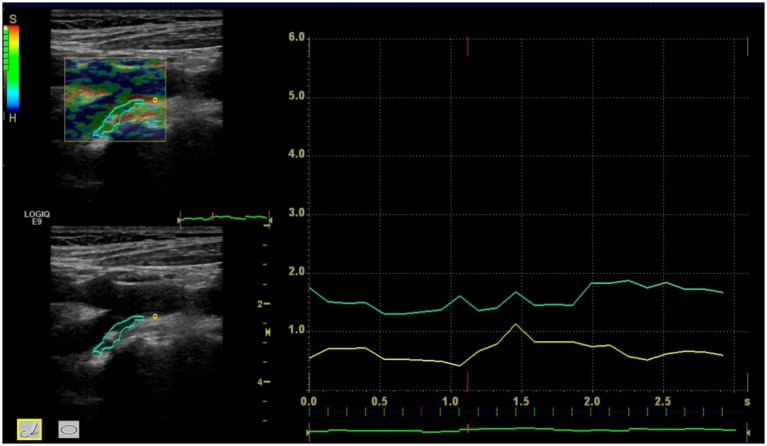
The carotid plaque strain elastography showed plaque strain elastography value and arterial wall strain elastography value.

Ultrasound strain elastography of carotid plaques revealed that plaques predominantly exhibited yellow-green or green color coding (Figure 1), while the arterial walls were predominantly coded as red. Comparison of plaque strain elastographic characteristics between the deterioration and non-deterioration groups after cerebral infarction demonstrated that statistically significant differences was found in carotid plaque strain elastography value and plaque stiffness (*p* < 0.05). However, no statistically significant difference was found in arterial wall elasticity value (*p* > 0.05). See Table 3 for details.

**Table 3:** Comparison of the strain elastographic characteristics of plaques between the deterioration group and the non-deterioration group.

Indicators	Deterioration group	Non-deterioration group	Test value	*p*-value
Number of cases	48	62		
Plaque strain elastography value	1.16 ± 0.40	1.72 ± 0.58	5.74	<0.001
Arterial Wall strain elastography value	0.53 ± 0.16	0.59 ± 0.18	1.95	0.053
Plaque stiffness	2.33 ± 0.97	3.04 ± 1.00	3.75	<0.001

### 3.4 Binary logistic regression analysis

Clinical progression after cerebral infarction (coded as 0 = no deterioration, 1 = deterioration) served as the dependent variable. Separate models were constructed for each independent variable: carotid plaque elasticity, vessel wall elasticity, and plaque hardness. The model assumptions, including linearity in the logit, were met. Binary logistic regression identified carotid plaque elasticity and plaque hardness as significant independent predictors of clinical progression (*p* < 0.05).

### 3.5 ROC curve analysis

**Figure 2:**
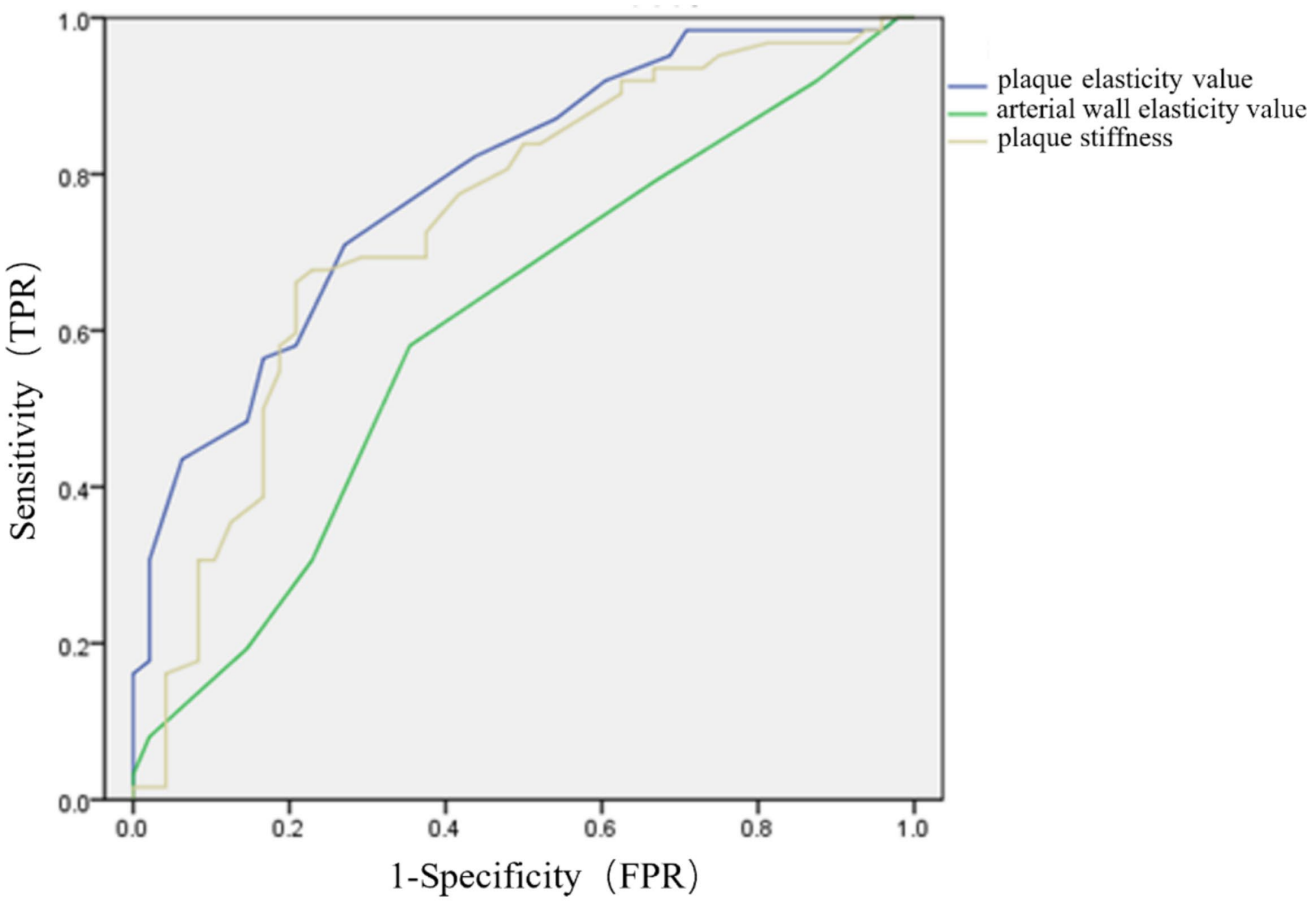
ROC curves for plaque elasticity value, arterial wall elasticity value and plaque stiffness in predicting clinical changes after cerebral infarction.

ROC curve analysis was leveraged to analyze the role of plaque elasticity value, arterial wall elasticity value and plaque stiffness in predicting clinical deterioration after cerebral infarction. The results indicated that the AUCs for plaque elasticity value, arterial wall elasticity value and plaque stiffness were 0.790 (95%CI 0.707–0.873), 0.608 (95%CI 0.501–0.715), and 0.740 (95%CI 0.644–0.835), respectively (Figure 2).

## 4 Discussion

Cerebral infarction is a leading cause of permanent disability, while vulnerable carotid plaques are recognized as a significant contributor to cerebral infarction (10, 11). Recent studies have identified common markers of plaque vulnerability—such as plaque thickness, size, and surface morphology—as being associated with the occurrence of ischemic cardiovascular and cerebrovascular events (12, 13). Routine ultrasound can determine the size, location, number, surface morphology of plaques and the degree of carotid stenosis, even the ulceration on the surface and bleeding within the plaques, but it has limited value in judging the nature of plaques (14, 15). Contrast-enhanced carotid plaque ultrasound provides information on intraplaque neovascularization, but is costly, time-consuming and carries the risk of allergies, limiting its clinical application (16). Ultrasound strain elastography, a recent ultrasound technique, has been widely used for organs such as the breast, thyroid and liver. Emerging evidence has suggested that the elastographic characteristics of plaques, including plaque stiffness, are associated with the occurrence of cerebral infarction, but few studies have investigated its potential in assessing clinical changes following cerebral infarction (17).

The basic principle of elastography is to apply an internal (including spontaneous) or external dynamic or static/quasi-static mechanical excitation to tissues. Under the physical laws of elasticity and biomechanics and so on. the tissues respond with certain changes in displacement, strain or velocity distribution, and so on. Common techniques include strain elastography and shear wave elastography, etc. (18) Studies have shown that soft plaques are lipid-rich, so they have low stiffness and high strain under compression, and mainly appear as yellow-green or green on elastographic imaging (19). In contrast, calcified plaques are lipid-poor and mainly composed of calcium, so they have high stiffness and low strain under compression and appear as blue. Mixed plaques are in between and present a mosaic pattern of blue and green.

In this study, patients with cerebral infarction who underwent carotid plaque strain elastography were classified into a deterioration group and a non-deterioration group based on clinical progression following infarction. Elastographic imaging showed that the deterioration group had lower plaque elasticity values and plaque stiffness than the non-deterioration group, with statistically significant differences. This indicates that patients with different changes in their condition after cerebral infarction have different carotid plaque elasticity values and plaque hardness. We can assess the risk of short-term deterioration of the patient’s condition through the results of carotid plaque elastography. This may be attributed to the fact that vulnerable plaques had a soft texture and were more prone to rupture, which can result in microthrombosis and persistent cerebral infarction. The microemboli detachment caused by vulnerable carotid plaques was also recognized as an independent risk factor for stroke progression, neurological impairment, and poor outcomes in patients with acute ischemic stroke (20, 21).

In this study, ROC curve analysis was leveraged to assess the predictive power of carotid plaque strain elastography value, arterial wall elasticity value, and plaque stiffness for clinical deterioration after cerebral infarction. The AUCs were 0.790, 0.608, and 0.740, respectively. These findings indicated that carotid plaque strain elastography held significant value in predicting clinical progression after cerebral infarction, especially in terms of plaque elasticity value. This provided important insights for clinical assessment of disease progression after cerebral infarction, followed by timely intervention when necessary.

However, due to inconsistent pressure applied, manual compression during elastography may introduce variability in the resulting strain and displacement measurements, thereby affecting the research results. In this study, the probe was gently placed over the carotid plaque and the pressure required for elastography was provided by the natural pulsations of carotid artery. This approach partly reduced operator-induced variability. All patients selected for this study showed no evidence of ≥50% stenosis in the intracranial or extracranial carotid artery, thereby reducing the impact of hemodynamics on the elastography results. This study analyzed the largest carotid plaque ipsilateral to the cerebral infarction. Although causation by this specific plaque cannot be confirmed, its characteristics reflect the patient’s overall atherosclerotic burden, providing useful information for clinical management. Given its single-center design, external validation via large-scale, multi-center studies is required. This analysis did not include a multivariable model to confirm if the relationship between plaque elastographic features and clinical deterioration is independent of covariates; addressing this limitation through a multimodal model will be our next research step.

## 5 Conclusion

In conclusion, carotid plaque ultrasound strain elastography provides a valuable adjunct for assessing plaque vulnerability. Patients experiencing clinical deterioration following cerebral infarction exhibit significantly lower plaque elasticity and lower plaque stiffness. Among these parameters, plaque elasticity value demonstrates enhanced predictive performance for post-cerebral infarction clinical deterioration. These findings may inform clinical intervention strategies for patients with cerebral infarction; however, multi-center studies with larger cohorts are required to validate the predictive utility of these parameters.

## Data Availability

The raw data supporting the conclusions of this article will be made available by the authors, without undue reservation.
